# Effect of a Low-Glycemic-Load Diet and Dietary Counseling on Acne Vulgaris Severity Among Female Patients Aged 15 to 35 Years

**DOI:** 10.7759/cureus.72886

**Published:** 2024-11-02

**Authors:** Qaisar Raza, Rashk-e Hina, Sadia Nawaz, Minahil Safdar, Kinza Imran, Urwah Ashraf, Muhammad Saeed Imran

**Affiliations:** 1 Department of Food Science and Human Nutrition, University of Veterinary and Animal Sciences, Lahore, PAK; 2 Department of Genetics, University of Veterinary and Animal Sciences, Lahore, PAK; 3 Department of Pathology, University of Veterinary and Animal Sciences, Lahore, PAK

**Keywords:** acne severity, acne vulgaris, dietary counseling, female patients, low-glycemic diet

## Abstract

Background: Acne is significantly influenced by glycemic load (GL), which is the result of the quantity of carbohydrates consumed and how quickly they are metabolized. There is an association between high-GL foods and severe acne. Such diets increase insulin and insulin-like growth factor 1 (IGF-1) levels, which then stimulate sebum production and androgen hormone release, which ultimately results in the development of acne. Previous studies have shown a high prevalence of acne in South Asia, but there is a lack of study on acne and its determinants among females in Pakistan.

Aim: This study aims to assess the effect of low-glycemic index foods and dietary counseling in potentially improving the quality of life of female acne patients and reducing post-acne lesions and the severity of the disease.

Methods: The sample size consisted of 50 females aged 15-35 years. The study aimed to see how a low-GL diet could affect the severity of acne. The trial included 50 participants, split into two groups: 25 in the control group and 25 in the treatment group. The trial went on for 12 weeks and used a food questionnaire to gather data each month. Participants were given scores based on how much high-GL food they ate. The score range between 0 and 35 showed a low intake of high-GL foods, and a score range between 35 and 55 was considered to be the average intake of high-GL foods. A score range between 55 and 75 showed a very high intake of high-GL foods. Higher scores indicated higher GL food consumption.

Results: In the control group, after one month, 18 participants (70%) had GL scores above 55, which correlated with higher acne severity. In contrast, only three participants (12%) had low-GL scores (0-35). The treatment group showed significant improvement; initially, 15 participants (60%) had high-GL scores, but this number decreased to nine participants (40%) by the end of the study. After three months, 10 participants (45%) in the treatment group achieved low-GL scores (0-35), compared to only two participants (10%) in the control group. This improvement was accompanied by a decrease in average acne severity from 2.68 to 1.56 and an increase in water intake and promoted better skin health among females.

Conclusion: Dietary counseling along with low-GL foods can improve acne severity and lesions in adult females. This treatment also enhanced self-confidence and social acceptance.

## Introduction

Acne is a very common and complex skin problem that badly affects individuals, especially adolescent females. Acne can result in considerable depression, frustration, anxiety, and healthcare costs. In many Western countries, acne affects 79-95% of the adolescent population, out of which 26% are females [1]. Dietary factors have long been considered in the pathogenesis of acne vulgaris. The relationship between diet and acne is of utmost interest in dermatology. It is an established fact that increased production of sebum plays an important role in the development and severity of acne, and evidence now suggests that dietary factors have an impact on the production of sebum from the sebaceous gland, as recent pieces of evidence reflect a re-consideration of nutritional aspects related to endocrine functioning, which is involved in acne development [2]. In the 1960s, acne was considered a disease caused by a disturbance of carbohydrate metabolism, and now studies support the fact that high carbohydrate intake increases the output of sebum and vice versa [3]. Recent studies demonstrate that the quantity, as well as composition of food, definitely affect acne pathogenesis. There is strong evidence that diet serves as a substrate for the production of sebaceous lipids [2]. Sebum produced by the human body consists of 40-60% of triglycerides, 19-26% of wax esters, and squalene, along with some cholesterol. Glucose and acetate produce these lipids, which synthesize sebum from glands [4]. The sebum that is produced is carried and released into the follicle of hair, from where it is then released on the skin surface. Anything that obstructs this pathway causes the accumulation of the material in that place, leading to the formation of acne vulgaris. Although acne is not caused by dietary factors alone, it does have a very critical and important role in the severity and longevity of the disease [5]. Many studies have demonstrated the importance of the glycemic load (GL) of foods in acne patients, showing that patients who have low-glycemic index (GI) foods in their daily diet have reduced acne lesions and post-acne percussions, as compared to those patients who have high-GL foods in their daily routine because high-GL carbohydrate intake is readily absorbed, resulting in high blood glucose and higher insulin production [6]. A diet that is high in GL is linked with an increased demand for insulin, which is needed to metabolize, and other problems related to insulin resistance, e.g., obesity. Higher insulin results in the higher production of hepatic insulin-like growth factors (IGF-1), which is linked with the increased production of sebum from the sebaceous glands, a major factor involved in the pathogenesis of acne [7]. It has been observed that individuals in Western countries who consume high-GL foods, including high amounts of refined carbohydrates, dairy products, processed foods, packaged items, etc., have a high prevalence of acne along with disturbed social life since acne also has detrimental psycho-social impacts [8].

In Bangladesh, the prevalence of acne is reported at 34.4%, a comparatively lower figure. In India, prevalence rates vary, with studies indicating 66.6% and 59.2% in different regions, whereas in Pakistan, the prevalence is estimated at approximately 55.9% [9-11]. In a study conducted in 2012 at Seoul National University Hospital, 32 participants with mild to moderate acne were given 10 weeks of dietary intervention, including a low-GL diet, which significantly improved the symptoms of acne and acne lesions. However, no significant change in the BMI was observed, probably due to the short study food group (the control group) and acne vulgaris, as the treatment group’s acne got better with a low-GL diet [12]. In another study conducted in 2013 in Iran where 70 women were included in the treatment group and 70 women were included in the control group, after 12 weeks of a low-GI diet (followed by the treatment group only), there was a significant positive relationship between high-GI diet and acne grade [13].

There is a lack of studies in Pakistan regarding acne treatment. Therefore, a randomized controlled trial study was conducted to study the effect of a low-GL diet and dietary counseling on acne vulgaris severity and lesions among female acne patients aged 15-35 years in Lahore.

## Materials and methods

Study design and location

This study aims to assess the effect of low-GI foods and dietary counseling in potentially improving the quality of life of female acne patients and reducing post-acne lesions and the severity of the disease. A randomized controlled trial was conducted to study the effect of a low-GL diet and acne vulgaris severity and lesions among female acne patients aged 15-35 years in Lahore, Pakistan, from October 2023 to December 2023. The study was conducted at two outlets of a famous derma clinic, namely Dr. Waris Anwar Aesthetics in Lahore, Pakistan, and Waris Clinic, Lahore, Pakistan.

Ethical statement

This study (number: 215/IRC/BMR) was approved by the Institutional Review Committee for Biomedical Research of the University of Veterinary and Health Sciences, Lahore, Pakistan. This Institutional Review Committee/Biomedical Research (IRC/BMR) research is undertaken in compliance with the guidelines set out by the Institutional Review Committee for Biomedical Research. Prior to the commencement of the study, all participants provided their informed consent to participate.

Study population

The study was conducted on 50 females aged 15-35 years. It was a 12-week program to evaluate the effect of high-GL foods on acne severity. The control group consisted of 25 participants (n1=25) and the same number of participants were allocated to the treatment group (n2=25). Participants in acne patients who received the dietary intervention (low-GL diet with counseling) and those in the control group (no dietary intervention). Participants excluded from the study were those currently taking any medication that is known to affect acne or glucose metabolism. Subjects who were taking any oral antibiotics or topical antibacterial, anti-acne, or retinoid agents and those who were under severe stress.

Data collection

Data was collected every month from the control group and treatment group, and for data collection, a food frequency questionnaire was used. The questionnaire consisted of different sections. The first section consisted of demographic data including the patient’s name, age, BMI (kg/m2), height (inches), weight (kilogram), and family income. To check dietary patterns, high-GL foods were added to the questionnaire, which were considered to be the primary foods playing a role in the development and severity of acne vulgaris. The foods mentioned in the questionnaire contributed to acne pathogenesis, which include fruits (mangoes, watermelon, red apple, banana), vegetables (carrots, potatoes, beetroot), milk and milk products, white bread/rice/pasta, sweets/desserts/ice cream, sugar added coffee, milk added coffee/tea, milk+sugar added coffee/tea, dry fruits (raisins, prunes, dates), sweet drinks (soft drinks, canned juice, soda, etc.), energy drinks, confectionaries (cookies, cakes, pastries, doughnuts, cake, brownies), milk chocolate, fast food (pizza, burger, fries, etc.), fried food items (samosa, pakoras, etc.). Questions about genetic history, water intake, and stress level were also added to know about the further association of any of these factors. To evaluate the effect, a scoring system was generated. The GL score was calculated from the questionnaire. Participants were given a score on the basis of their high-GL food intake. Those participants who were not taking any high-GL foods were given one score; those taking it 1-4 times per week were given two scores; participants taking high-GL foods once a day were given three scores; four scores were given if intake was 2-3 times per day; and those taking high-GL foods four or more times per day were given five scores. Hence, more scores indicated high-GL food intake and vice versa. A score range between 0 and 35 showed a low intake of high-GL foods. A score range between 35 and 55 was considered to be an average intake of high-GL foods. A score range of 55-75 showed a very high intake of high-GL foods. Shelley and Pillsbury gave the scoring system for acne vulgaris, which is as follows: Grade one: simple non-inflammatory acne - comedones and a few papules (elevated spot on the skin that is less than 1 cm). Grade two: comedones, papules, and a few pustules (inflamed and filled with pus spots on the skin). Grade three: large inflammatory papules, pustules, and a few cysts. Grade four: More severe inflammatory acne, with cysts becoming convergent [14]. Hence, every month, acne was graded depending on the skin condition, and the effect of a low-GL diet was evaluated.

Baseline measurements of acne grades were taken at the start of the study. Dietary counseling was given to the participants enrolled in the intervention group in the form of a low-GL diet plan. The diet plan consisted of fruits, vegetables, nuts, whole grains, and protein sources like fish and chicken. Weight, height, water intake, GL score, BMI category, and finally acne grade of participants in both the control and intervention groups were measured at baseline and after 30 days. Weight was measured in kg and height in inches, and BMI was calculated by dividing weight in kg with height in m^2^. Water intake in cups was recorded, and acne grade was determined depending on the number and size of inflammatory papules on the face and neck region after one month in both the control and intervention groups.

Statistical analysis

Descriptive analyses were carried out by using IBM SPSS Statistics for Windows, Version 22 (Released 2013; IBM Corp., Armonk, New York, United States). Baseline characteristics were analyzed using an independent sample t-test. To analyze the effect of dietary changes on acne severity, a paired sample t-test was utilized. This test is particularly useful when assessing changes within the same group at different times. Throughout the study's duration, alterations in acne severity were observed during follow-up visits for both the control group (participants not undergoing dietary interventions) and the treatment group (participants undergoing dietary interventions) over a span of three months. We compared changes in acne severity using paired sample t-tests at each follow-up visit of both the control group as well as the treatment group. The statistical significance of the findings was determined by analyzing p-values. In this study, a p-value less than 0.05 was considered significant, indicating that the observed changes in acne severity were likely due to the dietary intervention rather than random chance.

## Results

Changes in weight, GL score, acne grade, water intake, and BMI score after one month in both the control and intervention groups are shown in Table 1. Our main concern is the GL score and acne grade among both groups. In the control group, only 12% (3) of participants' GL scores were between 0 and 35, 18% (4) of participants' GL scores were between 35 and 55, and 70% (18) of the participants' GL scores were between 35 and 55, which was statistically associated with high 2 grade and 3-grade acne. While in the treatment group, only 15% (4) of the participants’ GL scores were between 0 and 35, 25% (6) of the participants' GL scores were between 35 and 55, and 60% (15) of the participants was between 35-55, which was statistically associated with a low percentage of grade 2 and grade 3 acne after one month of following a low-GL diet.

**Table 1:** Comparison of anthropometric measurements and acne grade in control and treatment group (after first month)

	Groups	Mean±SD		Change		p-value
		Before (1 month)	After (1 month)	N	%	
Weight (kg)	Control	64.28±2.88	63.76±2.87	-0.52	-0.81	0.12
	Treatment	69.68±6.93	68.28±6.65	-1.4	-0.02	0.00
BMI score	Control	25.29±2.40	25.20±2.50	-0.09	-0.35	0.77
	Treatment	25.88±2.92	25.37±2.79	-0.51	-2.01	0.00
Glycemic load score	Control	58.88±5.90	59.52±5.90	0.64	1.07	0.15
	Treatment	58.72±6.77	55.00±7.78	-3.88	-7.05	0.00
Water intake (cups)	Control	1.68±0.69	1.20±0.40	-0.48	-40	0.00
	Treatment	1.16±0.37	1.64±0.63	0.48	29.26	0.00
Acne grade	Control	3.04±0.45	3.08±0.40	0.04	1.29	0.32
	Treatment	3.04±0.20	2.92±0.40	-0.12	-4.10	0.18

Table 2 shows the change in weight, GL score, acne grade, water intake, and BMI score after the second month in both the control and intervention groups. In the control group, GL scores after the second month showed that 25% (6) of participants' GL scores were between 0 and 35, 20.22% (5) of participants' GL scores were between 35 and 55, and 54% (14) of the participants were between 35 and 55, which was statistically associated with even higher grade 2 and grade 3 acne, which showed that severity increases with the increase in GL score of the control group participants (n=25). In the treatment group, GL scores after the second month showed that 25% (6) of participants’ GL scores were between 0-35; 25% (6) of participants' GL scores were between 35 and 55; and 50% (13) of the participants were between 35 and 55, which was statistically associated with comparatively low grade 2 and grade 3 acne, as described in Table 2, after following a low-GL diet.

**Table 2:** Comparison of anthropometric measurements and acne grade in control and treatment group (after second month)

Parameters	Groups	Mean±SD		Change		p-value
		Before (2 months)	After (2 months)	N	%	
Weight (kg)	Control	63.76±2.87	63.68±6.93	-0.08	10.10	0.79
	Treatment	68.28±6.65	66.22±6.29	-2.06	27.50	0.00
BMI score	Control	25.20±2.50	24.20±2.50	1.00	12.30	0.00
	Treatment	25.37±2.79	24.64±2.84	+0.73	15.70	0.00
Glycemic load score	Control	59.52±5.90	59.52±5.92	0.00	10.40	1.00
	Treatment	55.00±7.78	50.20±7.23	-0.80	9.20	0.00
Water intake (cups)	Control	1.20±0.40	1.2800±0.44	0.008	3.20	0.53
	Treatment	1.64±0.63	3.16±0.63	1.76	20	0.00
Acne grade	Control	3.08±0.40	3.08±0.40	0.001	-6.3	0.32
	Treatment	2.92±0.400	2.68±0.40	-0.24	51	0.00

In the control group, GL scores after the third month showed that 10.02% (2) of participants' GL scores were between 0 and 35, 19.22% (5) of participants' GL scores were between 35 and 55, and 70.76% (18) of the participants were between 35 and 55, which was statistically associated with highest grade 2 and grade 3 acne, which showed that severity increases with the increase in GL score of the control group participants (n=25). In the treatment group, after dietary counseling regarding glycemic food intake and their effects on the body, GL scores after the third month showed that 45% (10) of participants’ GL scores were between 0 and 35, which was low-glycemic food intake, 15% (6) of participants' GL score was between 35 and 55, and 40% (9) of the participants' score was between 55 and 75, which showed a reduction in acne grade as well (Table 3).

**Table 3:** Comparison of anthropometric measurements and acne grade in control and treatment group (after third month)

Parameters	Groups	Mean±SD		Change		p-value
		Before (3 months)	After (3 months)	N	%	
Weight (kg)	Control	63.68±6.93	63.36±2.70	-0.32	-0.50	0.79
	Treatment	66.22±6.29	65.48±6.29	-0.74	-1.13	0.00
BMI score	Control	24.20±2.50	24.63±2.44	0.43	1.61	0.62
	Treatment	24.64±2.84	24.35±2.84	-0.29	-1.19	0.00
Glycemic load score	Control	59.52±5.92	60.48±6.35	0.96	1.58	1.02
	Treatment	50.20±7.23	39.16±7.23	-11.36	-29.60	0.00
Water intake (cups)	Control	1.28±0.44	1.36±0.49	0.08	5.88	0.53
	Treatment	3.16±0.63	3.68±0.63	0.52	14.13	0.00
Acne grade	Control	3.08±0.40	3.08±0.42	0.001	-0.02	0.32
	Treatment	2.68±0.40	1.56±0.40	-1.12	-71.70	0.00

## Discussion

This study aimed to examine the potential link between the consumption of high-glycemic-load foods and the prevalence of acne vulgaris among adult females residing in Lahore, Pakistan. The purpose of this study was to figure out the effectiveness of dietary counseling used as an intervention for acne-prone participants. The consumption of high-glycemic foods increases the likelihood of acne development in adult females, affecting overall quality of life and self-esteem within females. This study showed that high-glycemic foods contain easily available carbohydrates in the body, leading to the proliferation of acne in individuals. Previous research demonstrated a significant association between high-glycemic foods and the development of acne [15]. Another study states that a high GI and GL diet may stimulate acne proliferative pathways by influencing biochemical factors associated with acne in the past [16].

An important factor of our study was the dietary counseling of the participants and their guidance about food choices, which helped them to get better as skin plays a significant role in their prognosis of acne. It is established that dietary counseling is an integral part of any health outcome in every case and in our study, especially in managing acne on the skin. This study suggested that subjects with a low-GL diet resulted in improved inflammatory papules of acne vulgaris as compared to the control group through dietary counseling. Hence, this study showed similarity with previous studies in this regard where improved inflammatory markers have been observed with dietary interventions [17].

In this study, a link was seen between dietary medication with low-glycemic foods and acne, which closely resembles another study conducted in Melbourne among people of non-western backgrounds [18]. That study included 43 male acne patients aged 15-25 and its aim was to investigate the impact of a low-glycemic-load diet on acne lesions. Over a 12-week dietary intervention, the low-glycemic-load group demonstrated a more significant reduction in total acne lesion counts compared to the control group. Additionally, the experimental diet led to greater weight loss, improvements in body mass index, and enhanced insulin sensitivity. These findings suggest a potential link between nutrition-related lifestyle factors, acne, and insulin sensitivity.

There is evidence that a high-GL diet is linked closely to the development of acne and is responsible for post-acne consequences as well. There has been ongoing research in this area, and now evidence supports a strong connection between diet and acne. For example, in a study conducted in 2012 at Seoul National University Hospital, 32 participants with mild to moderate acne were given 10 weeks of dietary intervention, including a low-GL diet, which significantly improved the symptoms of acne and acne lesions. No significant change in the BMI was observed, probably due to the short study period, but the treatment group’s acne got better with the low-GL diet [13].

The implications of the results of this study are that it helped in understanding the management of acne and indicated that diet plays a significant role in the overall well-being of a person. It showed that diet and nutritional counseling are two major and important factors in managing chronic acne conditions in individuals. Thus, if the diet is managed properly, individuals could prevent acne conditions from occurring. We believe that our study has potentially contributed to the advancements in public health practice and general public awareness programs regarding the acne problem.

There are some limitations of this study that need to be addressed in the field of research. These limitations are small sample size and randomization. This study has been conducted on a very small portion of the population, and we can never generalize the results of this study on a huge population, but we can get some insights on this matter that diet does possess an important role in skin conditions, so for future, some more comprehensive study should be conducted on this field to get more generalized results for the population at a huge level.

## Conclusions

Acne treatment necessitates not only medicinal therapy but also dietary changes. Changes in food choices and habits can be highly beneficial because there are no negative effects like those described with medicinal therapy. The findings of this study revealed not only an improvement in the severity of acne vulgaris but also a reduction in acne lesions seen on various regions of the body, particularly the face and neck, as a result of reduced GL meal consumption in the treatment group. It also improved the inflammatory papules and the hydration condition. This leads to the conclusion that incorporating low-GL foods into the diet may reduce the severity of acne, decrease the number of lesions, and ultimately enhance overall quality of life.
